# Cytological and Morphological Analyses Reveal Distinct Features of Intestinal Development during *Xenopus tropicalis* Metamorphosis

**DOI:** 10.1371/journal.pone.0047407

**Published:** 2012-10-10

**Authors:** Job Sterling, Liezhen Fu, Kazuo Matsuura, Yun-Bo Shi

**Affiliations:** Section on Molecular Morphogenesis, Program in Cellular Regulation and Metabolism (PCRM), Eunice Kennedy Shriver National Institute of Child Health and Human Development (NICHD), National Institutes of Health (NIH), Bethesda, Maryland, United States of America; University Claude Bernard Lyon 1, France

## Abstract

**Background:**

The formation and/or maturation of adult organs in vertebrates often takes place during postembryonic development, a period around birth in mammals when thyroid hormone (T3) levels are high. The T3-dependent anuran metamorphosis serves as a model to study postembryonic development. Studies on the remodeling of the intestine during *Xenopus (X.) laevis* metamorphosis have shown that the development of the adult intestine involves de novo formation of adult stem cells in a process controlled by T3. On the other hand, *X. tropicalis,* highly related to *X. laevis,* offers a number of advantages for studying developmental mechanisms, especially at genome-wide level, over *X. laevis,* largely due to its shorter life cycle and sequenced genome. To establish *X. tropicalis* intestinal metamorphosis as a model for adult organogenesis, we analyzed the morphological and cytological changes in *X. tropicalis* intestine during metamorphosis.

**Methodology/Principal Findings:**

We observed that in *X. tropicalis,* the premetamorphic intestine was made of mainly a monolayer of larval epithelial cells surrounded by little connective tissue except in the single epithelial fold, the typhlosole. During metamorphosis, the larval epithelium degenerates and adult epithelium develops to form a multi-folded structure with elaborate connective tissue and muscles. Interestingly, typhlosole, which is likely critical for adult epithelial development, is present along the entire length of the small intestine in premetamorphic tadpoles, in contrast to *X. laevis,* where it is present only in the anterior 1/3. T3-treatment induces intestinal remodeling, including the shortening of the intestine and the typhlosole, just like in *X. laevis.*

**Conclusions/Significance:**

Our observations indicate that the intestine undergoes similar metamorphic changes in *X. laevis* and *X. tropicalis*, making it possible to use the large amount of information available on *X. laevis* intestinal metamorphosis and the genome sequence information and genetic advantages of *X. tropicalis* to dissect the pathways governing adult intestinal development.

## Introduction

The development of many vertebrate organs takes place in two steps, the formation of an immature but functional organ during embryogenesis followed by the maturation into the adult form. This second step often occurs during the so-called postembryonic development, a period around birth in mammals when plasma thyroid hormone (T3) concentrations are high [1] and involves the formation of organ/tissue-specific adult stem cells, such as the adult intestinal and hematopoietic stem cells [2]–[4]. The intestine is one such organ that has been well studied due to the continuous self-renewal of the intestinal epithelium, which is responsible for the food processing and nutrient absorption, throughout adult life in vertebrates [5]–[8]. This self-renewal is accomplished through stem cell divisions in the crypt, followed by their differentiation as the cells migrate along the crypt-villus axis and eventual death of the differentiated cells near the tip of the villus.

**Figure 1:**
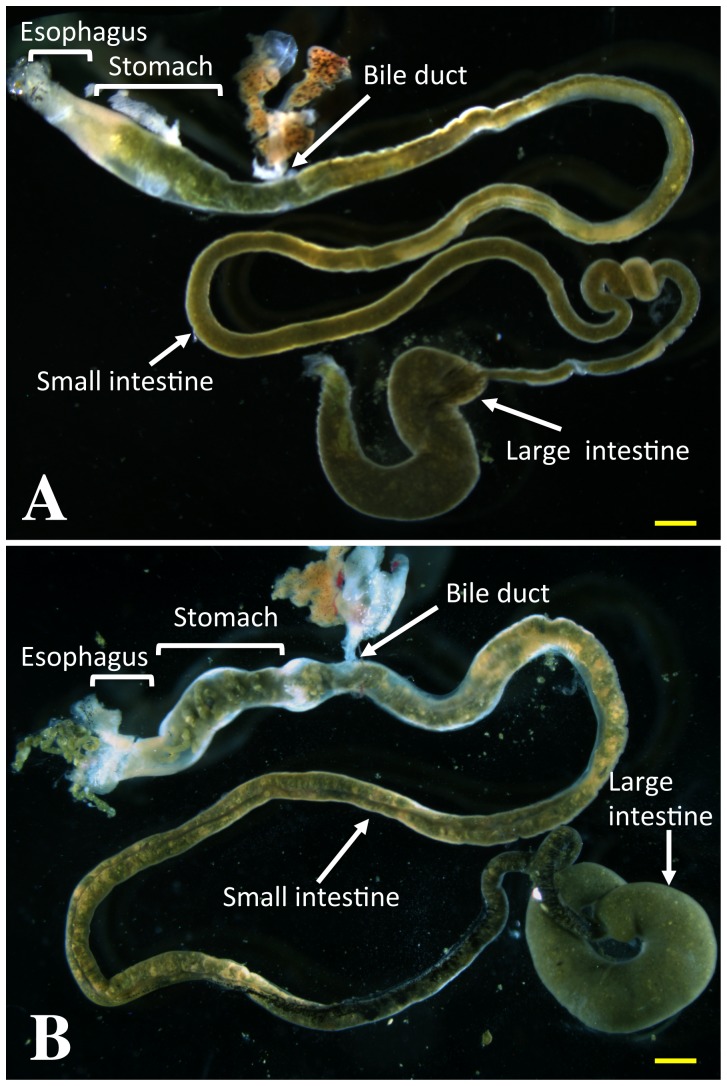
The gastrointestinal tract of a premetamorphic *X. laevis* (A) *and X. tropicalis* (B) tadpoles. Scale bars (yellow lines): 1 mm.

The establishment of the self-renewal system in the intestine takes place during postembryonic development in vertebrates. For example, in mouse, the intestinal epithelium lacks crypts at birth and the villus-crypt axis is formed in the first few weeks after birth as the plasma T3 concentration rises to a peak [2], [9], [10]. In amphibians such as *Xenopus (X.) laevis*, the transition from the tadpole/larval intestine to the adult form occurs during metamorphosis [2], [3], [8], [11], a process that shares many similarities with postembryonic development in mammals, including the requirement for high levels of T3 [1], [12]. The ability to easily manipulate amphibian metamorphosis has enabled extensive morphological, cellular, molecular, and genetic studies of intestinal remodeling during *X. laevis* metamorphosis. The tadpole intestine is made of mostly a monolayer of larval epithelial cells, with thin layers of connective tissue and muscles [11]. During metamorphosis, the larval epithelial cells undergo apoptosis and concurrently, adult epithelial stem/progenitor cells are formed de novo and rapidly proliferate [3]. Toward the end of metamorphosis, the adult epithelial cells differentiate to establish a trough-crest axis of epithelial folds, resembling the crypt-villus axis in the adult mammalian intestine, [2], [11].

Like other processes during metamorphosis, intestinal remodeling is totally dependent on T3 and can even be induced in cultures of isolated premetamorphic intestine upon T3 treatment [11]. Making use of these properties, we and others have shown that the adult epithelial stem cells are derived from the larval epithelium, likely through dedifferentiation of larval epithelial cells [13]–[15]. We have further shown that T3 can induce the formation of the progenitors or precursors of the adult epithelial stem cells tissue-autonomously while the formation of the stem cells also require the action of T3 in the surrounding tissues, most likely the underlying connective tissue [16]. In addition, many genes that are regulated during intestinal remodeling have also been identified by using microarray analyses and subtractive screening [17]–[20]. However, how these genes are regulated by T3 and what roles they play during adult stem cells development are largely unknown. This is in part due to some limitations of the *X. laevis* model. For example, *X. laevis* has a pseudo-tetraploid genome that has not been sequenced, making it difficult to carry out genome-wide studies such as ChIP (chromatin immunoprecipitation)-seq and ChIP-on-chip assays to study gene regulation mechanisms during development. In addition, it has a lengthy developmental cycle, which makes it difficult to study gene function during metamorphosis.

Using *Xenopus tropicalis*, which is highly related to *X. laevis*, can overcome these limitations [21], [22]. Its diploid genome has been sequenced and its developmental cycle is twice as fast as that of *X. laevis*. Furthermore, we and others have shown that the expression of T3 receptors (TRs) and 9-cis retinoic acid receptors (RXR), which form heterodimers to mediate the in vivo effects of T3 during *X. laevis* development [23]–[27], is similarly regulated during development of *X. tropicalis* and *X. laevis.* In addition, *X. tropicalis* TR/RXR heterodimers have expectedly similar transcriptional activities as *X. laevis* TR/RXR heterodimers [28]. Furthermore, ChIP analyses have shown that, as in *X. laevis,* TR is bound to two well studied TR target genes and induces histone modifications at the corresponding promoters in *X. tropicalis*, again resembling that in *X. laevis* [29], [30]. Thus, it is expected that T3 induces metamorphosis in *X. tropicalis* via similar molecular mechanisms as in *X. laevis*.

**Figure 2:**
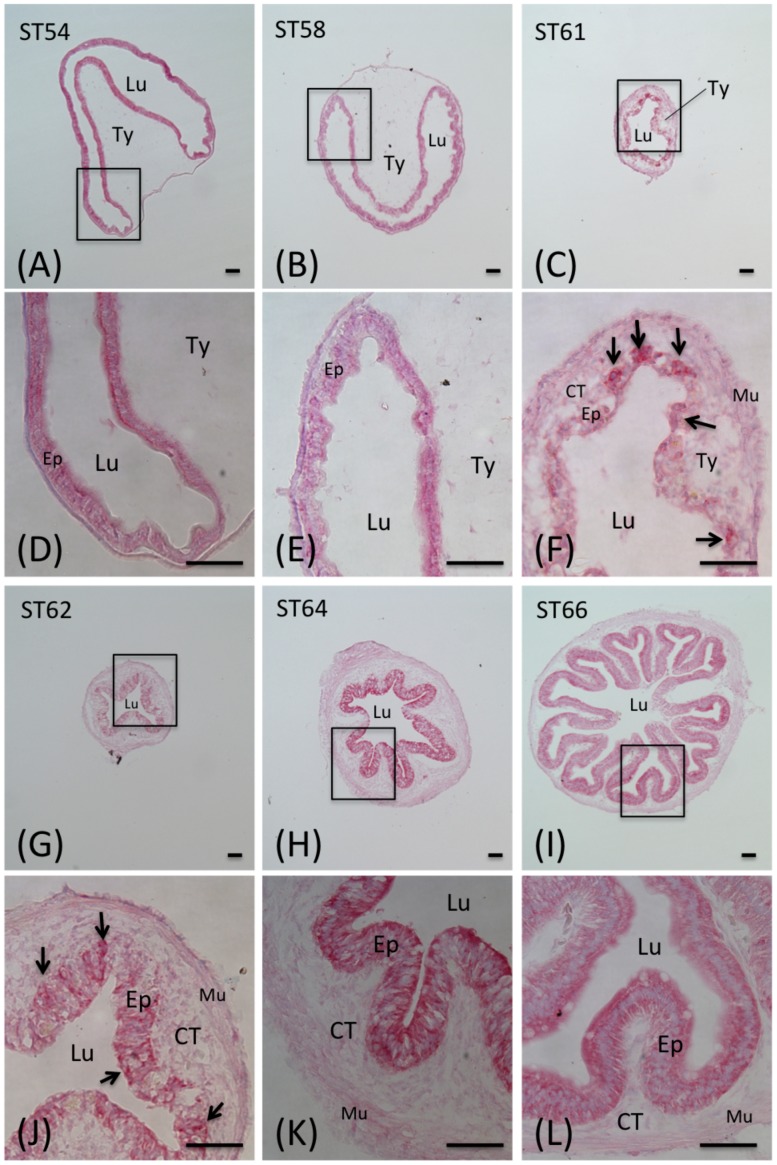
Morphological changes in the *Xenopus tropicalis* intestine during natural metamorphosis. The intestine of *Xenopus tropicalis* tadpoles at stage 54 to 66 was isolated and stained with MGPY and photographed with a light microscope. A–C and G–I: a cross-section of the intestine at the indicated stage. D–F and J–L: the enlarged photo of the boxed area in A–C and G–I, respectively. Note that during metamorphosis, the MGPY staining became weaker in larval epithelium as the cells undergo degeneration. At the climax of metamorphosis, the newly formed, proliferating adult epithelial islets were strongly stained by MGPY (arrows). The connective tissue (CT) and muscles (Mu) increased dramatically during metamorphosis. Ep: epithelium; Lu: lumen; Ty: typhlosole; Scale bars: 50 µm.

To investigate the possibility of using *X. tropicalis* as a model to study adult intestinal stem cell development, we have analyzed the morphological and cytological changes of the intestine during metamorphosis in *X. tropicalis*. Our results indicate that the metamorphic transformations in the intestine are conserved between *X. tropicalis* and *X. laevis*. We have also discovered a distinct difference, that is, the presence of the typhlosole throughout premetamorphic small intestine in *X. tropicalis* while only in the anterior 1/3 of the small intestine in *X. laevis*, suggesting possibly distinct regulation of the stem cell development along the anterior-posterior axis of the intestine.

**Figure 3:**
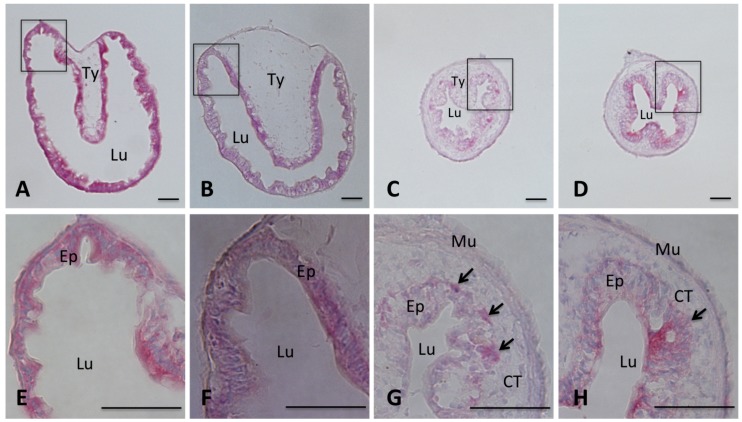
T3 induces intestinal remodeling in premetamorphic *X. tropicalis* tadpoles. Stages 54 *X. tropicalis* tadpoles were treated with 10 nM T3 at 25°C. The intestine was isolated from the tadpoles at day 0, 1, 3, and 5, respectively. The intestine was fixed, sectioned, and stained with MGPY as in Fig. 2. A–D: a cross-section of the intestine after 0, 1, 3, 5 days of T3 treatment, respectively. E–H: the enlarged image of the boxed area for the corresponding tissues in A–D, respectively. Note that after 3 days of T3 treatment, the MGPY staining became weaker in larval epithelium as the cells undergo apoptosis. At the same time, the newly formed, proliferating adult epithelial islets were strongly stained by MGPY (arrows). The connective tissue (CT) and muscles (Mu) increased dramatically during metamorphosis. Ep: epithelium; Lu: lumen; Ty: typhlosole. Scale bars: 50 µm.

## Experimental Procedures

### Animals

*Xenopus laevis* and *Xenopus tropicalis* tadpoles were either purchased from NASCO (Fort Atkinson, WI) or generated and reared in the laboratory. All experiments involving animals were performed following procedures approved by the Animal Use and Care Committee of Eunice Kennedy Shriver National Institute of Child Health and Human Development.

### T3 treatment and Methyl Green Pyronin Y (MGPY) Staining

Premetamorphic tadpoles at stage 54 were treated with 5 or 10 nM T3 at 18°C (*Xenopus laevis* tadpoles) or 25°C (*Xenopus tropicalis*) for indicated number of days. The gastrointestinal tract from *Xenopus laevis* and *tropicalis* tadpoles at different stages with or without T3-treatment were isolated and fixed in 4% paraformaldehyde overnight. They were then embedded in OCT compound and cryosectioned. The sections were frozen until further analysis.

**Figure 4:**
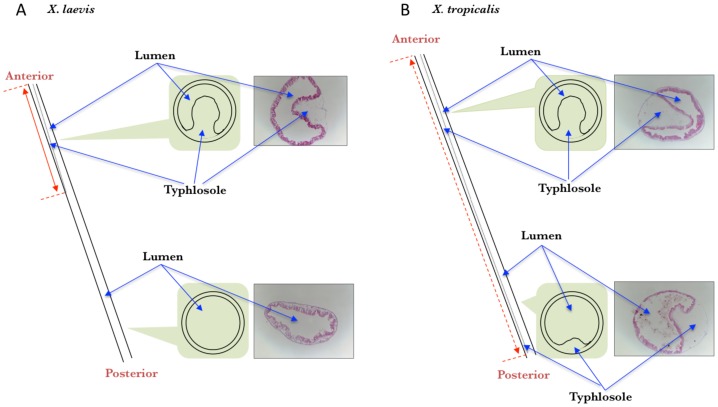
The typhlosole is present in the entire small intestine of premetamorphic *X. tropicalis* tadpoles (A) but only in the anterior small intestine of premetamorphic *X. laevis* tadpoles (B). A schematic diagram of the intestine from the anterior to the posterior is shown on the left (dashed lines indicate the boundaries of typhlosole with the intestines). On the right of each panel shows representative MGPY-stained cross-sections of the intestine from indicated regions of the intestine of premetamorphic tadpoles at stage 54. In the middle is a schematic drawing of the cross-section showing the presence or absence of the typhlosole. Note the presence of the typhlosole in the posterior half of the small intestine in *X. tropicalis* but not *X. laevis.*

For staining with MGPY, a mixture of methyl green, which binds strongly to DNA, and pyronin Y, which binds strongly to RNA [19], [25], [31], the slides were rinsed three times for 5 minutes each in 1X PBS, incubated in MGPY solution (Muto Pure Chemicals, Japan) for 15 min at room temperature, washed for 1 minute with 1X PBS, then rinsed in H_2_O, and finally dried on a slide warmer. The slides were subsequently incubated in 2 changes of Histoclear for 5 minutes each followed by mounting with a coverslip. The intestinal sections were analyzed with a light microscope and the imagines were recorded with a cool-coiled digital camera.

Terminal deoxynucleotidyl transferase dUTP nick end labeling (TUNEL) assay was performed essentially as previously described to label the apoptotic cell with Biotin-16-dUTP (Roche, IN) [32]. The Biotin-16-dUTP labeled apoptotic cells were visualized with Texas Red-Streptavidin staining (1∶200). The sections were counterstained with DAPI, washed with 1X PBS 3 times for 1 minute each and mounted with VECTASHIELD (VECTOR, Burlingame, CA). The intestinal sections were analyzed with a fluorescent microscope and the imagines were recorded with a cool-coiled digital camera at red fluorescent channel for Texas Red signal as well as blue fluorescent channel for DAPI, respectively, and merged to show the location of the apoptotic cells in the intestinal sections.

**Figure 5:**
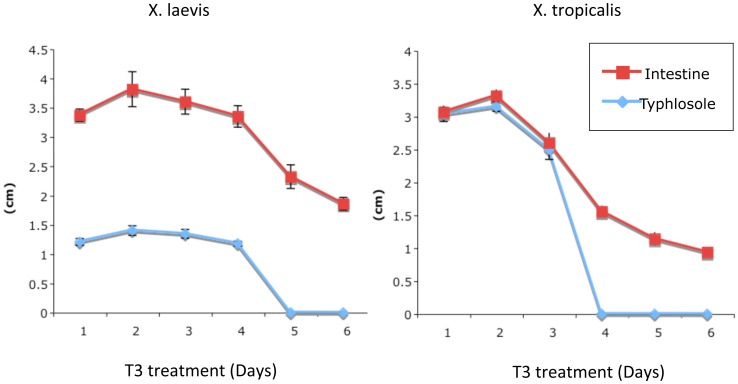
Reductions in the length of typhlosole and the small intestine of premetamorphic *X. laevis* and *X. tropicalis* tadpoles upon T3 treatment. Stage 54 *X. laevis* or *tropicalis* tadpoles were treated with 10 nM T3 at 18°C (*Xenopus laevis* tadpoles) or 25°C (*Xenopus tropicalis* tadpoles) for the indicated days and the intestine was isolated. The length of the typhlosole and the small intestine were measured. Note that the lengths of the intestine was reduced upon T3 treatment and that after 4–5 days of treatment, the typhlosole was no longer identifiable due to metamorphic changes in the intestine, resembling that at the climax of metamorphosis. The faster changes for *X. tropicalis* tadpoles were in part due to the higher temperature at which the animals needed to be reared.

### Intestinal Measurements

The entire tadpole intestine was dissected intact from tadpoles with or without T3 treatment and analyzed under a dissecting microscope to identify the boundary of the typhlosole in the intestine. The length of the entire intestine and the length of the typhlosole in the intestine were measured with a ruler. At least 3 tadpoles at each time point were measured. The mean and stand error were determined and presented.

**Figure 6:**
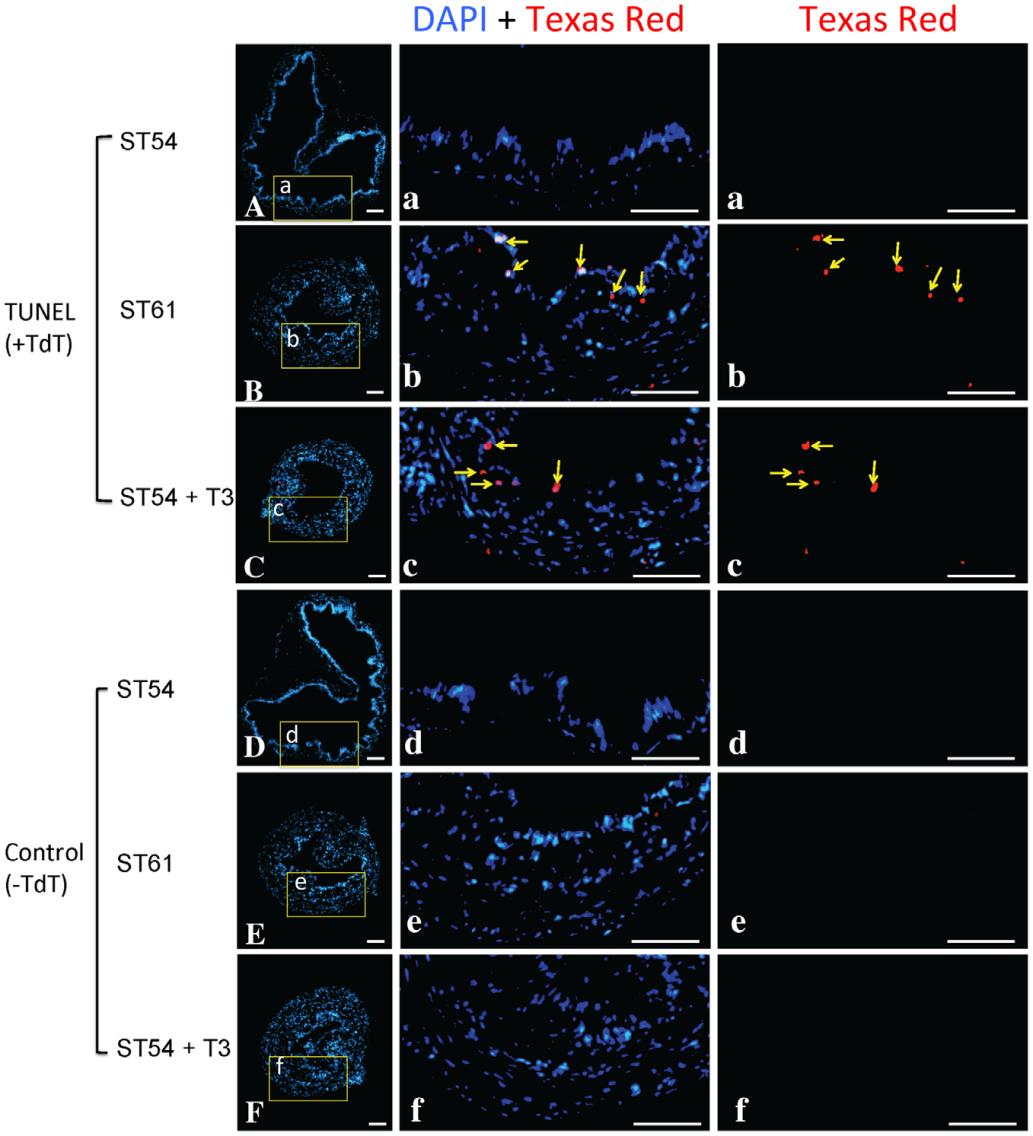
Intestinal epithelial apoptosis occurs during natural and T3-induced metamorphosis in *X. tropicalis*. The intestine from tadpoles at premetamorphic stage 54 (ST54), the climax of metamorphosis (ST61), or ST54 but treated with 5 nM T3 for 3 days (ST54+ T3), were analyzed by TUNEL assay (A–C) or TUNEL without the enzyme TdT as a the negative control (D–F). The biotin-dUTP labeled apoptotic cells were visualized with Texas-Red labeled Streptavidin and the nuclei were stained with DAPI. The boxed areas were shown at a higher magnification (a-f) with the apoptotic cells indicated with yellow arrows in either merged (DAPI+Texas Red) and single channel of red fluorescence (Texas-Red) images. Scale bar: 50 µm.

#### Quantitative reverse transcription-polymerase chain reactions (qRT-PCR)

Total RNA were extracted from intestines of *X. tropicalis* tadpoles during natural metamorphosis with TRIzol Reagent (Invitrogen, NY) and made DNA-free with DNase I treatment. The total RNA was reverse transcribed into cDNA as described previously [33] and subjected to qPCR analysis by SYBR® Green PCR (ABI, Foster City, CA). The primers used were 5¢-TTGCCAAGGTTGCTTTCCG-3′ and 5¢-GTTTCAGGATTGTGGGAGATAACG-3′ for the control gene rpl8 [34], 5¢-CGAGCCGGAGGCACAGACAAAG-3′ and 5¢-GCAGGTAGAAGGGTGAGGAGATGC-3′ for stromelysin 3 (ST3), as well as 5¢-CCATGGCCTTTGATGGAACC-3′ and 5¢-ATCTTCAGGTTGTCGTGTGCT-3′ for intestinal fatty acid binding protein gene (IFABP), respectively. The qPCR reactions were reproducibly repeated at least twice with three duplicates for each stage. The values for ST3 and IFABP were normalized against that of rpl8 and presented as means with standard deviations.

**Figure 7:**
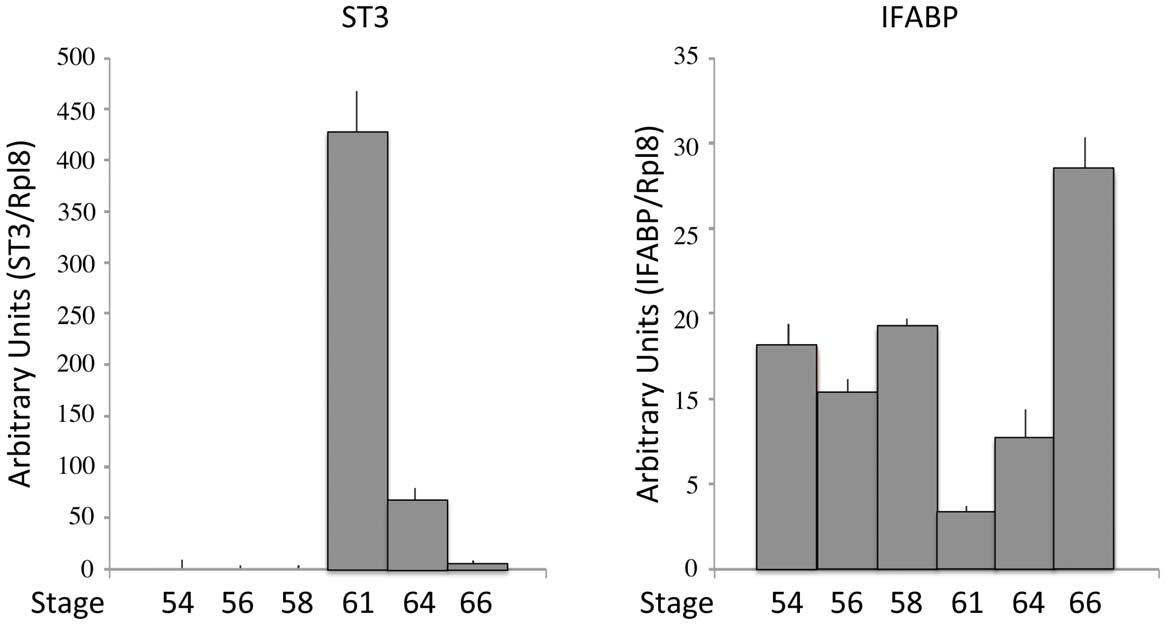
ST3 is upregulated while IFABP is downregulated during *X. tropicalis* intestinal metamorphosis. Total RNA were extracted from intestines of tadpoles at the indicated stages and analyzed by qRT-PCR. The expression levels for ST3 or IFABP were normalized against the control gene rpl8 and represented as means with with standard deviations in arbitrary units.

## Results

### The Metamorphosis of the Small Intestine in X. tropicalis Involves Degeneration of the Larval Epithelium Followed by the Formation of the New Adult Epithelium

The gastrointestinal tract in a premetamorphic *X. tropicalis* tadpole consists of the esophagus, stomach, small and large intestine as in other vertebrates (Fig. 1). Like in premetamorphic *X. laevis* tadpoles, the small intestine is very long and is the largest part of the gastrointestinal tract.

To examine the changes in the intestine during metamorphosis, cross-sections of the anterior small intestine from *X. tropicalis* tadpoles at different stages of metamorphosis were stained with MGPY, which distinguishes the proliferating adult epithelial cells from the dying larval epithelial cells based on their RNA content [19], [25], [31]. As shown in Fig. 2, at premetamorphic stage 54, the luminal side of the intestine was a monolayer of larval epithelial cells that was stained strongly and uniformly by MGPY (Fig. 2A/D). The epithelium was surrounded by thin layers of connective tissue and outer muscles. There was a single epithelial fold, the typhlosole, where connective tissue was abundant. This structure remained largely unchanged through the onset of metamorphic climax (stage 58, Fig. 2B/E). By the climax of metamorphosis (stages 60–64), the intestine had undergone extensive changes. At stage 61 (Fig. 2C/F), the larval epithelium became very weakly stained by MGPY due to the degeneration of the larval cells, leading to reduced RNA in the cells; at the same time the clusters of newly formed adult epithelial progenitor/stem cells were strongly stained, just like those observed in *X. laevis* [19], [25], [31]. By stage 62, the adult epithelium began to fold and the typhlosole was no longer observable (Fig. 2G/J). From stage 64–66, the epithelium developed into the multiply folded adult structure (Fig. 2H/K, I/L). All these mimic those during *X. laevis* metamorphosis.

### T3 Induces Metamorphic Changes in the Small Intestine in X. tropicalis Premetamorphic Tadpoles

T3 treatment of premetamorphic *X. laevis* tadpoles is known to induce similar changes in the intestine as during natural metamorphosis. To investigate T3-induced changes in the intestine of *X. tropicalis* tadpoles, we treated stage 54 tadpoles with T3 for 0–5 days and the intestine was isolated and cross-sections of the anterior small intestine were stained with MGPY. As shown in Fig. 3, after 0 or 1 day of T3 treatment, the intestinal epithelium was stained uniformly and there were little connective tissue and muscles except in the typhlosole where the connective tissue was abundant (Fig. 3 A, B/E, F). After 3 days of treatment, most of the larval epithelium became weakly stained as the larval cells degenerated and clusters of newly formed adult cells were strongly stained (Fig. 3C/G), resembling the intestine at stage 61 (Fig. 2 D/H). After 5 days, most of the epithelium was stained well and adult epithelial folds began to form and the typhlosole was no longer recognizable (Fig. 4D/H), similarly to the intestine at stage 62 (Fig. 2 I/L). The connective tissue and muscles developed extensively by the end of the treatment. Thus, T3-induces intestinal remodeling mimics that during natural metamorphosis.

### The Typhlosole is Present Along the Entire Length of the Small Intestine of Premetamorphic X. tropicalis Tadpoles

The typhlosole is an interesting and important structure of the intestine in premetamorphic *X. laevis* tadpoles [11], [35]. It is present along the anterior one third of the small intestine [36] and is the only location where the connective tissue is abundant (Fig. 4A). As shown above and in Fig. 4B, the anterior intestine of premetamorphic *X. tropicalis* tadpoles also had the typhlosole. Interestingly, when we examined the posterior part of the small intestine in premetamorphic *X. tropicalis* tadpoles, we were surprised to find that it too had the typhlosole (Fig. 4B). In fact, we observed that the typhlosole was present along the entire length of the small intestine in premetamorphic *X. tropicalis* tadpoles as diagramed in Fig. 4.

### T3 Treatment of premetamorphic Tadpoles Leads to the Reduction in the Length of Both the Small Intestine and the Typhlosole

During metamorphosis, the small intestine reduces its length by as much as 90% in *X. laevis* and the T3 treatment also causes reduction in the length of the small intestine [11]. To compare how the length of the intestine and typhlosole changes in *X. laevis* and *X. tropicalis,* stage 54 premetamorphic tadpoles of both species were treated with 10 nM T3 for 0–6 days and the length of the intestine and typhlosole was measured. As shown in Fig. 5, the length of the intestine began to decrease after 3 or 5 days of T3 treatment in *X. laevis* or *X. tropicalis* tadpoles, respectively. The length of the typhlosole was the same as the small intestine up to 3 days of T3 treatment in *X. tropicalis* and after 4 days of treatment or longer, the typhlosole was no longer identifiable as the newly formed adult epithelial folds were no longer distinguishable from the typhlosole (Fig. 3D). In *X. laevis,* the length of the typhlosole were maintained at 1/3 of the length of the small intestine up to 4 days of T3 treatment and after 5 days of T3 treatment or longer, the typhlosole was no longer identifiable. Thus, T3 treatment induced similar changes in the lengths of the typhlosole and the small intestine in both *X. laevis* and *X. tropicalis.* The only difference between the two species in response to T3 was that the changes were faster in *X. tropicalis* than in *X. laevis*. While it is unknown if the different organizations of the typhlosole in the two species contribute to the their different response to T3, this might be due, at least in part, to the faster development of *X. tropicalis* tadpoles and/or the fact that *X. tropicalis* tadpoles need to be reared at higher temperature, thus responding to T3 faster.

#### T3 induces larval epithelial degeneration through apoptosis and gene regulation in the intestine of X. tropicalis tadpoles

*X. laevis* larval intestinal epithelium degenerates through apoptosis during both natural and T3-induced metamorphosis, accompanied by the changes in the expression of many genes [25], [26], [33]. To explore if *X. tropicalis* intestinal epithelium undergoes similar changes, we performed TUNEL assay on cryosections of intestines of stage 54 tadpoles (premetamorphic tadpoles), stage 61 tadpoles (climax of metamorphosis), and stage 54 tadpoles treated with T3 for 3 days, in the presence (Fig. 6A–C) or absence of TdT, the enzyme essential for TUNEL assay (Fig. 6D–F). The results showed that apoptotic cells were rarely detectable in the intestines of stage 54 tadpoles (Fig. 6A, a) but abundant in the intestinal epithelia of stage 61 tadpoles (Fig. 6B, b) and that of stage 54 tadpoles treated with T3 for 3 days (Fig. 6C, c), similar to those observed in *X. laevis*.

In addition, we examined the expression of two genes known to be regulated by T3 in *X. laevis*: stromelysin-3 (ST3), which encodes a matrix metalloproteinase and known to play a causative role during *X. laevis* intestinal epithelial apoptosis [33], [37], and intestinal fatty acid binding protein (IFABP), which is a differentiation marker down-regulated at the climax of metamorphosis in the *X. laevis* intestine [38], in the intestines during *X. tropicalis* metamorphosis (Fig. 7). Just like in *X. laevis*, ST3 was up-regulated to reach the highest level while IFABP was down-regulated to the lowest level at stage 61 (Fig.7), the climax of metamorphosis when larval epithelia were undergoing apoptosis (Fig.6B &b) and adult epithelial progenitor/stem cells were developing (Fig. 2C & F).

## Discussion and Conclusion

Adult organ-specific stem cells are critical for organ homeostasis, tissue repair and regeneration. These stem cells are often developed during postembryonic vertebrate development, a period around birth in mammals when T3 levels are high. It has been difficult to study postembryonic development in mammals due to the nature of the in utero development. Anuran metamorphosis resembles the postembryonic development in mammals but occur independent of parental influence. It is totally dependent on T3 and can be easily manipulated by controlling the availability of T3 in the tadpoles. Studies on *X. laevis* metamorphosis have revealed extensive molecular mechanistic details on the regulation of this process by T3, including the role of T3 receptors and many genes that are regulated by T3 during metamorphosis [2], [39]. Of many changes during metamorphosis, intestinal remodeling offers a unique opportunity to study the development of the adult organ-specific stem cells [2], [40]. In *X. laevis,* this process involves the degeneration of the larval epithelium through apoptosis or programmed cell death [11]. Concurrently, the adult epithelial stem or progenitor cells develop de novo through dedifferentiation of some cells in the larval epithelium, followed by the proliferation and differentiation to form a multiply folded adult epithelium surrounded by elaborate connective tissue and muscles, resembling that in adult mammals. Interesting, this transition appears to be conserved in mammals as mouse adult intestinal stem cells are also formed during postembryonic development in a process that involves similar regulation by T3 [40]–[43]. Thus *X. laevis* intestinal remodeling offers an opportunity to manipulate and study adult stem cell development in vertebrates. On the other hand, its lengthy developmental cycle and its yet-to-be sequenced pseudo-tetraploid genome make it difficult to study gene function and genome-wide analyses in *X. laevis.* Here our studies suggest that intestinal remodeling in *X. tropicalis* resembles that in *X. laevis* and thus offers an alternative model for studying intestinal development without the aforementioned drawbacks.

*X. tropicalis* is highly related to *X. laevis* but has a simple diploid genome that has been sequenced [21], [22]. It also has a shorter life cycle, requiring only half the time to reach sexual maturity from fertilization than that in *X. laevis.* This advantageous shorter life cycle coupled with its smaller body size makes the *X. tropicalis* a superior model for genetic and genome-wide functional studies. Importantly, our analyses here have shown that intestinal metamorphosis in *X. tropicalis* resembles that in *X. laevis*. Furthermore, our present work and limited earlier studies on T3 receptor (TR) function and regulation of T3 target genes have also demonstrated conserved function and mechanisms for TR during *X. tropicalis* metamorphosis [28]–[30], [44], [45]. Thus, one can combine the advantages of the *X. tropicalis* model with the wealth of cellular and molecular knowledge gained from studies on *X. laevis* intestinal metamorphosis in the past few decades to dissect the molecular pathways governing adult stem cell development during metamorphosis.

While the metamorphic changes in the intestine are similar between *X. laevis* and *X. tropicalis*, a major structure difference was observed between these two related species. A distinct feature of the premetamorphic *X. laevis* tadpole intestine is the presence of the typhlosole in the anterior 1/3 of the small intestine [36]. This is where the connective tissue is abundant in the tadpole intestine prior to metamorphosis. During metamorphosis, as adult epithelial folding occurs with the development of the connective tissue and muscles around the climax of metamorphosis (around stage 62), the typhlosole is no longer observable or distinguishable from the many newly formed epithelial folds. While the physiological significance of the typhlosole in premetamorphic tadpoles is unclear, in vitro organ culture studies suggest that it may play a role in the development of the adult intestinal stem cells. When fragments of the anterior small intestine with typhlosole were isolated from premetamorphic *X. laevis* tadpoles and cultured in vitro with T3, intestinal metamorphosis, including apoptosis of larval epithelial cells and development of the adult epithelium, took place [46]. On the other hand, when fragments of the small intestine lacking typhlosole were isolated from premetamorphic *X. laevis* tadpoles and cultured in vitro with T3, only larval epithelial degeneration was observed [47], suggesting that the connective tissue rich typhlosole is critical for adult epithelium development. Consistently, we have recently shown that expression of a constitutive active TR in the larval epithelium alone (resembling T3 treatment of only the epithelium) in typhlosole-containing organ cultures led to larval epithelial cell death and adult epithelial progenitor/precursor formation, however, such cells failed to express intestinal stem cell markers or develop into differentiated adult epithelium [16]. In contrast, when the constitutive active TR was expressed in all tissues of the intestinal fragment, which contained the typhlosole, in organ cultures, larval cell death, adult stem cell formation, and eventually adult epithelium development took place. These findings suggest that T3 action in the larval epithelium alone can induce the formation of adult progenitor/precursor cells while T3 action in the non-epithelium, most likely the connective tissue underlying the epithelium, is also required for these progenitors/precursors to become stem cells, presumably by forming or enabling the formation of the stem cell niche. Thus, it is interesting to speculate that the presence of the connective tissue-rich typhlosole along the entire length of the small intestine may facilitate the concurrent development of adult intestinal stem cells throughout the intestine during metamorphosis. It would be interesting to test this possibility in the future by using organ culture of intestine isolated from anterior as well as posterior small intestine of *X. tropicalis* premetamorphic tadpoles.
